# Artificial intelligence-driven clinical guideline recommendations in maternal care: How trustworthy are they?

**DOI:** 10.7705/biomedica.7902

**Published:** 2025-12-10

**Authors:** Jairo J. Pérez, Andrés F. Giraldo-Forero, Santiago Rúa, Daniel Betancur, Zuliany Urquina, Pablo Castañeda, Sara Arango-Valencia, Juan Guillermo Barrientos-Gómez, Ever A. Torres-Silva, Andrés Orozco-Duque

**Affiliations:** 1 Departamento de Ciencias Aplicadas, Instituto Tecnológico Metropolitano, Medellín, Colombia Instituto Tecnológico Metropolitano Departamento de Ciencias Aplicadas Instituto Tecnológico Metropolitano Medellín Colombia; 2 Facultad de Ingeniería, Instituto Tecnológico Metropolitano, Medellín, Colombia Instituto Tecnológico Metropolitano Facultad de Ingeniería Instituto Tecnológico Metropolitano Medellín Colombia; 3 Escuela de Ciencias Básicas, Tecnología e Ingeniería, Universidad Nacional Abierta y a Distancia, Bogotá, D. C., Colombia Universidad Nacional Abierta y a Distancia Escuela de Ciencias Básicas Tecnología e Ingeniería Universidad Nacional Abierta y a Distancia Bogotá, D. C. Colombia; 4 Facultad de Ingeniería, Institución Universitaria de Envigado, Envigado, Colombia Institución Universitaria de Envigado Facultad de Ingeniería Institución Universitaria de Envigado Envigado Colombia; 5 Dirección Científica, Clínica Universitaria Bolivariana, Medellín, Colombia Clínica Universitaria Bolivariana Dirección Científica Clínica Universitaria Bolivariana Medellín Colombia; 6 Escuela de Ciencias de la Salud, Universidad Pontificia Bolivariana, Medellín, Colombia Universidad Pontificia Bolivariana Escuela de Ciencias de la Salud Universidad Pontificia Bolivariana Medellín Colombia

**Keywords:** Clinical guidelines as topic, maternal healthcare services, artificial intelligence, large language models, natural language processing., guías como asunto, servicios de salud materna, inteligencia artificial, modelos de lenguaje a gran escala, procesamiento de lenguaje natural.

## Abstract

**Introduction.:**

Medical staff often face difficulties in consulting and applying clinical guidelines in practice. Large language models, especially when combined with retrieval- augmented generation, may help overcome these challenges by producing context-specific outputs with improved adherence to medical guidelines.

**Objectives.:**

To assess the performance of commercial large language models in answering maternal health questions within retrieval-augmented generation systems, using both human and automated evaluation metrics.

**Material and methods.:**

A controlled experiment was designed to obtain accurate, consistent answers from a retrieval-augmented generation system based on Colombian maternal care guidelines. A physician formulated ten questions and defined the ground- truth answers. Various large language models were tested with a standardized prompt and evaluated through binary answer-concept ranking and retrieval-augmented generation assessment, metrics, judged by two independent large language models.

**Results.:**

Generative pre-trained transformer 3.5 (GPT-3.5) achieved the highest physician- assessed accuracy (0.90). Claude 3.5 obtained the top faithfulness score (0.78) under GPT-4.0 evaluation, while Mistral ranked highest (0.84) under Claude 3.5 evaluation. Regarding answer relevance, GPT-3.5 scored highest across both judges (0.94 and 0.86).

**Conclusions.:**

Integrating retrieval-augmented generation into obstetric care has the potential to enhance evidence-based practices and improve patient outcomes. However, rigorous validation of accuracy and context-specific reliability is essential before clinical deployment. The findings of this study indicate that large-scale models (*e.g*., GPT-3.5, Claude, Llama 70B) consistently outperform lighter models such as Llama 8B.

## Resumen

**Introducción.:**

El personal médico enfrenta limitaciones al consultar y utilizar guías clínicas en la práctica. Las recientes tecnologías de inteligencia artificial, como los modelos de lenguaje a gran escala -también llamados “pesados- (*large language models*, LLM), pueden ayudar a superar estas limitaciones. Cuando se usa la generación aumentada por recuperación (*retrieval-augmented generation*, RAG) a estos modelos, las respuestas generadas se vuelven más relevantes en contextos específicos y se ajustan mejor a las guías médicas.

**Objetivo.:**

Evaluar el desempeño de los modelos comerciales de lenguaje a gran escala mediante sus respuestas, cuando se trata de preguntas relacionadas con la atención materna en sistemas de generación aumentada por recuperación, supervisados estos sistemas mediante mediciones humanas y automáticas.

**Material y métodos.:**

Se diseñó un experimento controlado para obtener respuestas precisas y constantes de un sistema de generación aumentada por recuperación, utilizando las guías colombianas para la atención materna. Un médico formuló diez preguntas y determinó las respuestas de referencia. Se generó una instrucción (*prompt*) para la inteligencia artificial y se evaluaron varios modelos de lenguaje a gran escala, utilizando: 1) una clasificación binaria humana de conceptos en las respuestas, y 2) los valores de la evaluación de la generación aumentada por recuperación (*retrieval-augmented generation assessment*, RAGAS), juzgadas por otro modelo de lenguaje.

**Resultados.:**

El modelo GPT-3.5 (*generative pre-trained transformer* 3.5) obtuvo la puntuación más alta en la evaluación médica, con una precisión de 0,9. Mediante la valoración por GPT-4, el modelo Claude 3.5 fue el mejor calificado, destacándose una fidelidad de 0,78. Por otra parte, mediante la evaluación por Claude 3.5, el modelo Mistral obtuvo la puntuación más alta, con una fidelidad de 0,84. En cuanto a la relevancia de las respuestas, el modelo GPT-3.5 obtuvo la puntuación más alta en ambas evaluaciones: 0,94 con GPT-4o y 0,86 con Claude 3.5.

**Conclusiones.:**

La integración de la generación aumentada por recuperación en obstetricia puede mejorar las prácticas basadas en la evidencia, optimizando los resultados para los pacientes. Sin embargo, es crucial evaluar la precisión de las respuestas y la información específica del contexto antes de su uso clínico. Los resultados del presente estudio sugieren que los modelos pesados o a gran escala, como GPT-3.5, Claude o Llama 70B, superan siempre a los modelos más livianos o a menor escala, como Llama 8B.

The rates of maternal complications and deaths related to healthcare remain a major public health concern. In line with the third United Nations’ Sustainable Development Goal, the target for 2030 is to reduce the maternal mortality rate to fewer than 70 cases per 100,000 live births. Although global progress has been achieved, disparities persist between high- and low-income countries. In 2020, the maternal mortality rate in low-income countries, such as those in Latin America, was 430 per 100,000 live births, compared to 12 per 100,000 live births in high-income countries ^1^. According to the Colombian *Instituto Nacional de Salud*, the country reported an estimated maternal mortality rate of 43.8 per 100,000 live births in 2024. However, in six territorial entities, it exceeded 100 per 100,000 live births, highlighting significant regional inequalities ^2^.

Pregnancy-related complications demand precision and speed in clinical decision-making. Hypertensive disorders-including gestational hypertension, preeclampsia, and eclampsia- as well as obstetric bleeding and obstructed labor are among the leading causes of maternal mortality in Latin America ^3^. Other common pregnancy complications, such as gestational diabetes mellitus, preterm labor, depression, anxiety, and pregnancy loss, also require continuous monitoring. Optimizing clinical practices is therefore crucial to enhancing maternal healthcare and reducing preventable complications ^4^.

Medical guidelines are fundamental tools that support clinical decisionmaking by providing evidence-based recommendations reviewed by methodological and clinical experts. In Colombia, a multidisciplinary team coordinated by the *Ministerio de Salud y Protección Social,* developed the *Guías de práctica clínica para la prevención, detección temprana y tratamiento de las complicaciones del embarazo, parto o puerperio*^5^, hereafter referred to as the Colombian guidelines. This resource provides valuable and reliable information for maternal and fetal care, addressing disease-specific management, diagnostic technologies, maternal health promotion, and prenatal care.

While clinical guidelines are essential for standardizing care, reviewing such extensive documents can be complex and time-consuming. Physicians often face time constraints which hinder routine consultation and implementation. To overcome these limitations, technological solutions are being explored to facilitate the integration of guidelines into clinical workflows ^6^. Particularly, automated clinical decision support systems that feature guideline-based feedback are of growing interest, as they can improve efficiency and reduce costs for healthcare institutions ^7^.

Recent advances in artificial intelligence have introduced large language models, which perform remarkably as natural language chatbots. As conversational agents, these models can process large volumes of medical information and provide rapid and accurate answers to human questions. Retrieval-augmented generation further enhances their context-specific applicability by combining document retrieval with response generation. The retrieval step identifies relevant documents or information from a large dataset based on the user’s query. The generation step synthesizes this information into a detailed and accurate response. For example, when a user submits a question, the system retrieves the most relevant guideline content and generates an answer aligned with it.

In other words, retrieval-augmented generation enables large language models to produce outputs consistent with standard recommendations ^8^. Unlike models that rely solely on pretrained knowledge or general internet data, this type of model equipped with such system can generate answers grounded in specific medical guidelines. However, successful integration into clinical practice requires content validation, as reliability remains a concern. Thus, continued physician oversight becomes critical to ensure that outputs are clinically sound and adhere to best practices ^9^.

Xiong *et al.* described the development and evaluation of one of these models equipped with this generation system designed for preoperative medicine, demonstrating promising alignment with established clinical guidelines ^10^. Similarly, Macia *et al.* proposed an architecture based in retrieval-augmented generation to enhance the adoption of guidelines within the United Kingdom’s National Health Service, leveraging the large language model-as-a-judge framework and highlighting the need to correlate this model’s ratings and human ratings ^11^. Kresevic *et al*., for their part, introduced a large language model correlated with retrieval-augmented generation framework incorporating hepatology guidelines and compared the model-generated outputs with the expert answers ^12^. To date, however, no known studies have validated the retrieval-augmented generation systems using Spanish-language clinical guidelines for maternal care ^13^.

The objective of this study was to conduct a preliminary evaluation of commercial large language models in the context of maternal healthcare. Specifically, this work aims to:

1. establish a performance ranking of large language models and provide insights into the applicability of retrieval-augmented generation for maternal care using Spanish-language clinical guidelines;
2. assess the effectiveness of retrieval-augmented generation systems through an open-source evaluation framework that measures the quality of large language models outputs against clinical guidelines, and
3. explore future research directions within a retrieval-augmented generation framework, using prompt engineering and targeted questions to rigorously assess the scalability and generalizability of these models in healthcare settings ^14^.

Accordingly, and in alignment with the TRIPOD-LLM statement, this study focuses on large language models evaluation: “Assessing or testing an existing LLM to determine its efficacy, accuracy, or suitability for a specific task within healthcare” ^15^.

## Materials and methods

A controlled experiment was conducted using the *Guías de práctica clínica para la prevención, detección temprana y tratamiento de las complicaciones del embarazo, parto o puerperio*. To evaluate the performance of a retrieval-augmented generation architecture in answering questions relevant to this guideline, a dataset comprising ten questions and ten corresponding ground-truth answers was constructed (table 1). The ground truth was defined by a physician with four years of experience at a reference institution for high-risk obstetrics, which provides highly complex gynecology and obstetrics services. The physician ensured complete adherence to the guideline in defining the answers, thereby reducing subjectivity and minimizing potential variability.

**Table 1.** Questions on prenatal care used for large language models evaluation (English translation included for consistency)

No	Translated question [English]	Original question [Spanish]
1	When and by whom should the clinical risk of a patient with a normal pregnancy be reassessed?	¿En qué momento y quién debe reevaluar el riesgo clínico de una paciente con embarazo de curso normal?
2	What is the ideal gestational week to start prenatal check-ups?	¿Cuál es la semana gestacional ideal para iniciar los controles prenatales?
3	When is the start of prenatal check-ups considered late?	¿Cuándo se considera tardío el inicio de los controles prenatales?
4	What is the ideal number of prenatal check-ups?	¿Cuál es el número ideal de controles prenatales?
5	When should psychosocial risk assessment be performed during maternal follow-up?	¿Con cuál herramienta se debe valorar el riesgo psicosocial en los controles prenatales?
6	Which tool should be used to perform psychosocial risk assessment in prenatal check-ups?	¿Cuándo se debe valorar el riesgo psicosocial en el seguimiento de una materna?
7	How often should postpartum depression screening be performed during pregnancy?	¿Cada cuánto debe hacer el tamizaje para la depresión posparto durante el embarazo?
8	What is the probability of vaginal delivery after a cesarean section?	¿Cuál es la probabilidad de un parto vaginal después de una cesárea?
9	¿Cuáles son las metas de ganancia de peso en las mujeres gestantes?	What are the recommended weight gain goals for pregnant women?
10	What is the recommended treatment for nausea and vomiting during pregnancy?	¿Cuál es el tratamiento recomendado para las náuseas y el vómito durante el embarazo?

Seven commercial large language models randomly selected and accessed via application programming interfaces (API) in a Python-based retrieval-augmented generation system, were tested. The code used in the experiments is publicly available at https://github.com/andresgiraldo3312/Maternal_care_RAG. The models were run sequentially in the order presented in table 2.

**Table 2.** Characteristics of the large language models included in the evaluation

Model	Size	Description	Available
Claude 3.5	≈ 175 B	Anthropic language model from the Claude 3.5 series, optimized for advanced natural language processing with improved reasoning and contextual comprehension.	AWS Bedrock [16]
Mistral	123 B	Mistral AI model from the ‘Large’ series, designed for high-performance text generation and reasoning capabilities.	AWS Bedrock [17]
Llama 3	8 B	Instruction-tuned version of the Meta AI model, optimized for following instructions and generating text with improved alignment.	AWS Bedrock [18]
Llama 3	70 B	Instruction-tuned version of the Meta AI model, designed for advanced NLP tasks, code generation, and deep reasoning.	AWS Bedrock [19]
Llama 3.1	8 B	Model optimized for multilingual dialogue and open-source applications, trained with fine-tuning and reinforcement learning with human feedback.	Ollama [20]
GPT-3.5	≈ 175 B	OpenAI model optimized for real-time text generation with improved computational efficiency.	OpenAI [21]
GPT-4o	≈ 200 B	OpenAI model designed for advanced NLP tasks, with enhanced contextual depth and reasoning capabilities.	OpenAI [22]

### Retrieval-augmented generation system configuration

**Figure 1.**
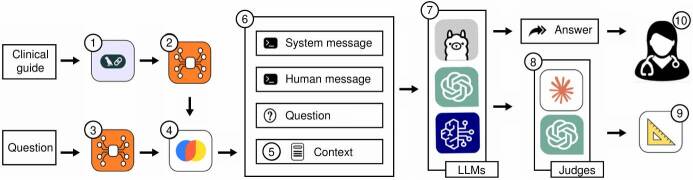
Retrieval-augmented generation system workflow. (1) Text chunking from clinical guideline; (2) Chunk embedding; (3) Vector database creation; (4) Question embedding; (5) Similar chunk retrieval; (6) Prompt design; (7) Large language model execution; (8) Automated answer evaluation; (9) Metric computation; and (10) Physician-led binary assessment

Access to relevant sources is critical for large language models to provide accurate answers to user questions based on clinical guidelines. These sources must ensure that data are peer-reviewed and that recommendations are evidence-based, thereby maintaining reliability of generated outputs. Table 3 summarizes the tools used to build and evaluate the system, while figure 1 presents the experimental framework, which consists of the following ten steps:

**Table 3.** Tools used to build and evaluate the retrieval-augmented generation system

Icon	Description	Icon	Description
**Figure:** 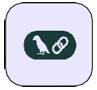	LangChain is a framework designed to support the development of applications that integrate large language models [23].	**Figure:** 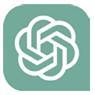	OpenAI large language models accessed via application programming interfaces (e.g., GPT-3.5 and GPT-4o).
**Figure:** 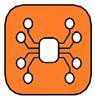	The embedding model converts text into numerical vectors that capture semantic meaning [24].	**Figure:** 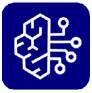	Large language models available on the Amazon Bedrock platform accessed via application programming interfaces (e.g., Llama 3.1).
**Figure:** 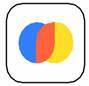	Chroma is an open-source vector database for efficient storage and retrieval of embeddings [25].	**Figure:** 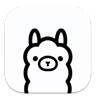	Ollama is a lightweight framework for running large language models locally on personal machines [26].
**Figure:** 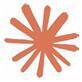	Anthropic large language models accessed via application programming interfaces (e.g., Claude 3.5).	**Figure:** 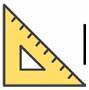	Retrieval-augmented generation assessment is an evaluation framework for retrieval-augmented generation pipelines [27].

1. Text was extracted from the clinical guideline’s PDF file using the LangChain framework. The extracted content was then segmented into 1,920 chunks, each containing 1,024 characters with an overlap of 200 characters, as recommended in the literature ^28^.
2. Each chunk was mapped to a 384-dimensional dense vector space using the all-MiniLM-L12-v2 embedding model from Hugging Face.
3. The resulting vectorized chunks were stored in Chroma, a vector database commonly employed for retrieval in augmented generation systems.
4. The questions listed in table 1 were embedded using the same model as in step 2.
5. For each question, the five most similar chunks (top-k) were retrieved using cosine similarity.
6. A prompt was designed by combining the system message, the human message, the question, and the context, as outlined in table 4.
7. Answers were generated by running large language models through the Ollama framework, Bedrock, and OpenAI application programming interfaces.
8. Answers were first evaluated with retrieval-augmented generation assessment, which relies on large language models acting as judges.
9. From this evaluation, quantitative performance metrics were obtained.
10. Binary answer-concept ratings were provided by physicians, as briefly explained below.

**Table 4.** Example of prompt composition (original version in Spanish)

System message
Eres un especialista en ginecología y obstetricia, te vas a comportar como un asesor para responder algunas preguntas de las guías clínicas.
Human message
A continuación, se presenta una pregunta seguida de documentos relevantes. Proporcione una respuesta detallada basada en estos documentos. Si la respuesta no está en los documentos, responda: “La respuesta no está en la guía”.
Question
¿En qué momento y quién debe reevaluar el riesgo clínico de una paciente con embarazo de curso normal?
Context
El seguimiento del embarazo es un proceso continuo que evoluciona gradualmente hasta el momento del parto. La evidencia muestra que el riesgo debe evaluarse lo más temprano posible, idealmente, en una consulta preconcepcional o en la primera consulta de control prenatal antes de la semana 10. Esta detección precoz permite intervenir algunos riesgos que son modificables, tales como enfermedades infecciosas o trastornos metabólicos, entre otros. Los estudios incluidos muestran múltiples factores biológicos y…
… el embarazo temprano o que estén embarazadas inmediatamente después de la vacunación pueden estar seguras, ya que no se ha documentado ningún caso de síndrome de rubeola congénita. Las mujeres que desean concebir deben ser aconsejadas y remitidas a una consulta preconcepcional, para determinar su estado inmunológico y vacunarlas contra la rubeola, si es necesario.
**Table 4.** Example of prompt composition (translated version in English)
System message
You are a specialist in gynecology and obstetrics and will act as an advisor to answer questions based on clinical guidelines.
Human message
Below is a question followed by relevant documents. Please provide a detailed answer based on these documents. If the answer is not contained in the documents, respond: “The answer is not in the guideline.”
Question
When and by whom should the clinical risk of a patient with a normal pregnancy be reassessed?
Context
Pregnancy monitoring is a continuous process that evolves gradually up to the moment of delivery. Evidence shows that risk should be assessed as early as possible, ideally during a preconception consultation or the first prenatal visit before the 10th week. Early detection enables intervention in modifiable risks such as infectious diseases or metabolic disorders. The included studies highlight multiple biological factors...
... Women who become pregnant early or immediately after vaccination can be reassured, as no cases of congenital rubella syndrome have been documented. Women wishing to conceive should be advised and referred to a preconception consultation to determine antibody status and, if necessary, be vaccinated against rubella.

It is worth mentioning that the system message defined the role and task of the model, while the human message instructed that the answer be based exclusively on the provided context. The context corresponded to the guideline chunks most relevant to the question ^29^.

This experimental design enabled consistent responses from a retrieval-augmented generation system configured with Colombian clinical guidelines and a structured prompt ^14^. Performance was assessed using two complementary methods: retrieval-augmented generation assessment metrics and the binary answer-concept ranking.

### Binary answer-concept ranking

All answers were reviewed by the previously mentioned physician, trained in obstetrics and gynecology, considering three aspects: the ground-truth answer (the literal answer from the guidelines), the context (the human and system messages provided in table 4), and the physician’s own clinical judgment. Each answer was scored in binary form, where 1 indicated “Correct” and 0 indicated “Not correct”. After scoring the ten answers, the values were summed to yield a total between 0 and 10. These totals were then normalized to a 0 to 1 range, converted into an index, and presented in a bar graph labeled *Accuracy.*

### Retrieval-augmented generation assessment

All answers were evaluated using retrieval-augmented generation assessment metrics, which focus on four dimensions: faithfulness, answer relevance, context recall, and context precision ^27^. To automate evaluation and ensure consistency, two large language models were chosen as judges: GPT-4o (judge 1) and Claude 3.5 (judge 2). Both models scored the answers across the four metrics, each measured on a 0 to 1 scale.

*Faithfulness* assesses whether the answer generated is factually consistent with the context provided. An answer is considered faithful if all claims can be inferred from the context. Scores closer to 1 indicate full consistency, ensuring that answers are reliable and grounded in the evidence.

*Answer relevance* measures how well the generated answer addresses the user’s original prompt. Incomplete or redundant answers receive lower scores, while complete and accurate answers score higher. This metric is computed using cosine similarity between the original user input and a set of artificially generated questions derived from the answer, thus assessing alignment with user intent.

*Context recall* determines the proportion of relevant documents or fragments successfully retrieved, focusing on minimizing omissions. It is computed by comparing retrieved results against a reference set. Higher recall values indicate fewer omissions of relevant content.

*Lastly, context precision* quantifies the proportion of retrieved fragments that are relevant, reflecting the accuracy of retrieval.

### Statistical analysis of retrieval-augmented generation assessment metrics

The statistical consistency and distributional properties of the retrieval-augmented generation assessment metrics were analyzed. Data were uploaded to the Posit Cloud environment and processed in R. The four variables-faithfulness, answer relevance, context recall, and context precision-were tested for randomness using the Runs Test and for normality using the Shapiro-Wilk test. Variables that violated these assumptions were analyzed with the Kruskal-Wallis test, whereas normally distributed variables were assessed with one-way ANOVA to determine statistical significance.

In addition, averages were computed at two levels: the overall average score per model across all metrics and the average score per metric across models. While these averages are not metrics themselves, they provide informative summaries for comparing model performance and understanding metric-specific behavior. This dual perspective -model-wise and metric-wise- facilitates alignment between human evaluation and retrieval-augmented generation assessment metrics, helping to identify top-performing models and highlight strengths or weaknesses in specific metrics.

### Ethical considerations

The use of large language models in retrieval-augmented generation applications for extracting information from clinical guidelines offers substantial value in terms of efficiency and decision-making. However, several clinical risks and ethical challenges must be addressed. Although such models can provide evidence-based recommendations, final decisions must remain with specialists, as large language models lack the ability to fully interpret patient-specific clinical contexts. The risk of misleading or inaccurate outputs persists, as models may generate information that, while grounded in guidelines, may not be directly applicable to individual cases.

From an ethical standpoint, the use of these models also raises concerns regarding patient data privacy, particularly when information is processed in uncontrolled environments. Therefore, it is essential to ensure that retrieval- augmented generation systems function strictly as support tools rather than substitutes for human judgment, with robust security protocols in place to protect sensitive information, while consistently respecting both the autonomy of healthcare professionals and the patient confidentiality.

## Results

The models presented in table 2 were evaluated using retrieval-augmented generation assessment. The answers were tabulated, and the models’ performance was analyzed as follows.

### Models’ performance based on binary answer-concept ranking

**Figure 2.**
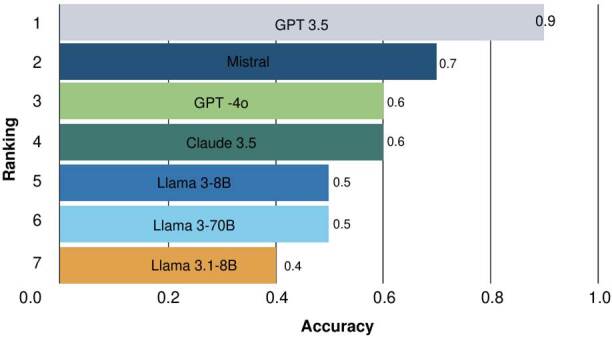
Ranking of models based on binary answer-concept accuracy

Figure 2 shows the evaluation of each answer generated by the models, assessed through human judgment. GPT-3.5 achieved the highest proportion of correct answers, with an index of 0.9, outperforming the others by at least 0.2. In contrast, lightweight models such as Llama 3.1-8B showed the lowest proportion of correct answers. This marked difference highlights a performance gap between large-scale generative models, with hundreds of billions of parameters, and mid-sized or compact models or variants such as the Llama 3 family.

Human evaluators generally favored the more natural answers from GPT- 3.5, even when they were not always the most strictly faithful to the source. For their part, Mistral, Claude 3.5, and GPT-4o performed moderately, with scores ranging between 0.6 and 0.5. Llama 3 did not surpass 0.5, which suggests lower accuracy in this scenario compared to the top-ranking GPT models. These results indicate the need to reinforce heavier models, such as Mistral, to improve their suitability for clinical applications.

### Models’ performance based on retrieval-augmented generation assessment metrics

Table 5 presents the results of the retrieval-augmented generation assessment metrics evaluated by the two selected judges. An asterisk (*) indicates cases in which a model acted as its own judge, a condition that may introduce bias by potentially inflating performance estimates. The results support the observation that mid-sized models and their variants underperform compared to larger-scale models, particularly in terms of answer relevance. For instance, Llama 3.1 and Llama 3 achieved the lowest scores for this metric.

**Table 5.**
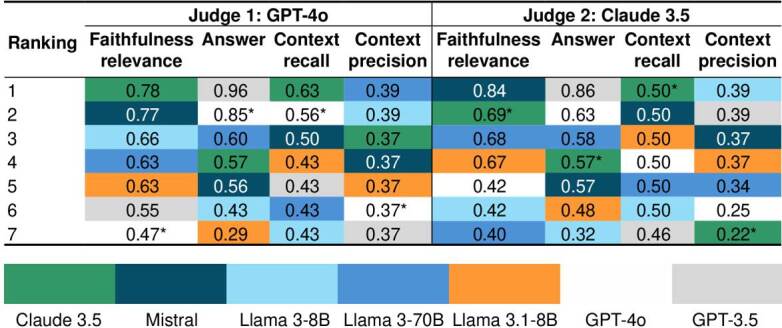
Comparison of model performance across evaluation metrics, ranked from the highest to the lowest score according to judge 1 (GPT-4o) and judge 2 (Claude 3.5)

For the purposes of this application, *faithfulness* was defined as alignment with the source guidelines, answer relevance as the appropriateness of responses; *context recall* as the ability to retrieve correct information, and *context precision* as accuracy in contextual interpretation. Both judge 1 and judge 2 applied similar parameters, enabling direct comparison of their evaluations.

In general, heavy models such as Claude 3.5, Mistral, and GPT-3.5 received higher scores in *faithfulness* and *context recall.* Conversely, Llama 3.1 -8B and Llama 3-70B obtained relatively lower scores across several metrics, reflecting limitations in following context and delivering precise answers, likely related to their smaller size and reduced reasoning capacity. The evaluations conducted by GPT-4o and Claude 3.5 highlighted *answer relevance* as the best-performing metric, with scores of 0.96 and 0.86, respectively.

Overall, larger models consistently demonstrated higher *faithfulness* and *answer relevance*, confirming that heavy architecture generates more consistent and reliable information. In contrast, none of the models achieved strong performance in context-related metrics, supporting the hypothesis that advanced architecture and training improve the quality of answers without necessarily ensuring robust context retention.

The *context precision* metric directly reflects the efficiency of the retriever in supplying accurate and relevant information, similar to the *context recall* metric. According to judge 1 and judge 2, *context recall* remained relatively consistent across most models (0.43-0.63, approximately). Claude 3.5 showed the lowest *context precision* (0.22), indicating difficulties in providing precise contextual information.

These observations are critical for understanding how each model improves its answers based on the quality of the information retrieved. Notably, *context precision* values should be highlighted, as they are very similar across models due to the search algorithm retrieving nearly identical content chunks using cosine similarity when assembling the context window. In contrast, the other metrics display greater variability, as they are more directly influenced by reasoning complexity and model capabilities.

**Figure 3.**
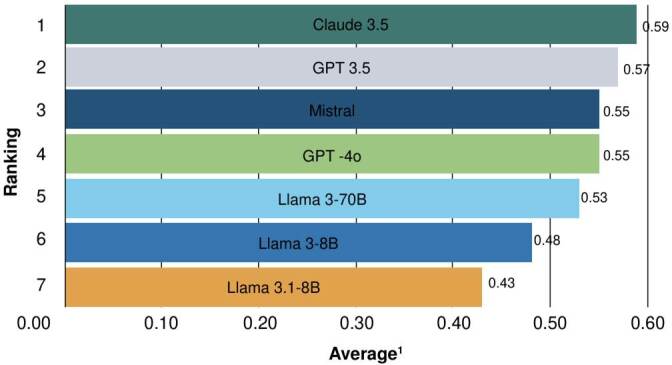
Retrieval-augmented generation assessment ranking of models based on average scores by judge 1 (GPT-4o)

**Figure 4.**
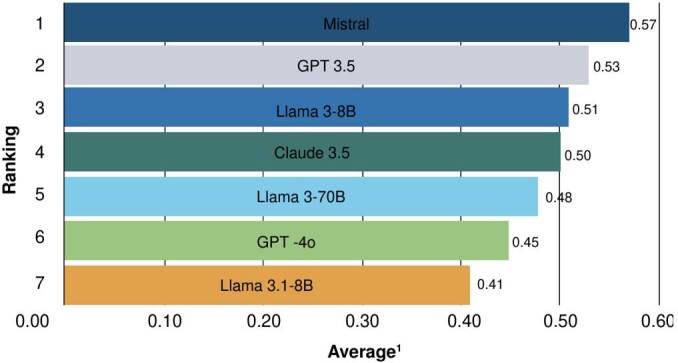
Retrieval-augmented generation assessment of models based on average scores by judge 2 (Claude 3.5)

The measures from judge 1 and judge 2 were averaged and presented in figures 3 and 4. According to judge 1, Claude 3.5 achieved the highest average (0.59), while Mistral ranked best forjudge 2 (0.57). GPT-3.5, for its part, yielded consistent outcomes across both evaluations. Conversely, Llama 3.1-8B again exhibited the lowest averages (0.43 and 0.41) for the two judges.

**Figure 5.**
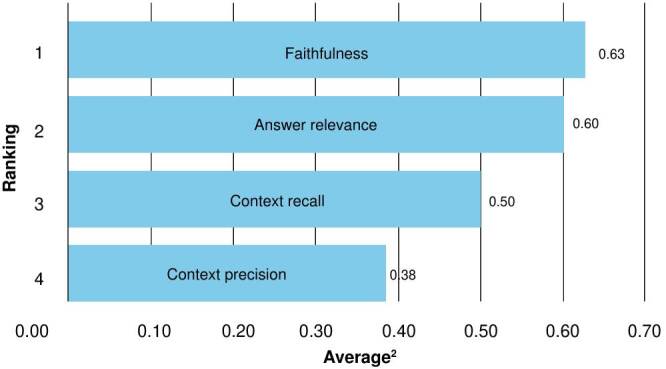
Retrieval-augmented generation assessment ranking of metrics averaged across models by judge 1 (GPT-4o)

**Figure 6.**
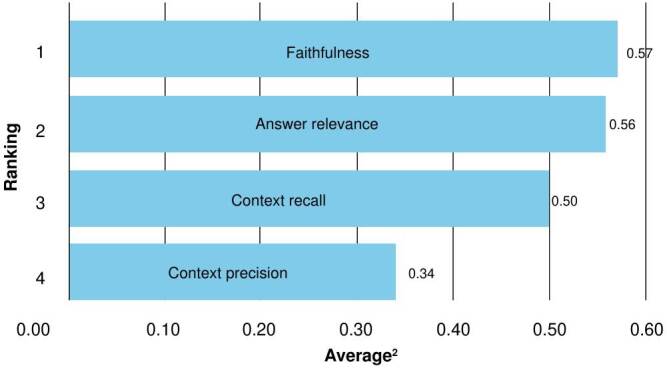
Retrieval-augmented generation assessment ranking of metrics averaged across models by judge 2 (Claude 3.5)

Figures 5 and 6 illustrate the average results of each assessment metric across all models. In general, the weakest-performing metrics were *context recall* and *context precision*, suggesting the need to improve retriever performance.

### Statistical analysis of retrieval-augmented generation assessment metrics

*Answer relevance, faithfulness*, and *context precision* violated at least one assumption (randomness or normality); therefore, the non-parametric Kruskal-Wallis test was applied. None of these three variables showed significant differences between judges (p = 1 for all three). By contrast, *context recall* met both assumptions and was analyzed using a one-way ANOVA, which also indicated no significant differences (p = 1).

Given the limited sample size (n = 14), it is important to acknowledge that these tests may lack sufficient statistical power, increasing the likelihood of type II errors, where true effects remain undetected due to insufficient data.

## Discussion

This study suggests that retrieval-augmented generation-based artificial intelligence models can support the application of clinical guidelines for maternal care. However, their performance varies according to model scale. Overall, large-scale models such as Claude 3.5, GPT-3.5, and Mistral, outperformed mid-scale models and their smaller variants, such as Llama 3.1 -8B. The ranking derived from human evaluations aligns with the retrieval- augmented generation assessment *answer relevance* metric, where GPT-3.5 performed best. GPT-4o and Claude 3.5 also stood out, while smaller-scale models consistently obtained the lower scores.

As shown in figure 5, *faithfulness* and *answer relevance* were among the highest scoring metrics. Nevertheless, models such as GPT-3.5 and GPT-4o, which achieved strong results in *answer relevance*, did not perform as well in *faithfulness*. This discrepancy may stem from their tendency to prioritize fluency, coherence, and relevance over precision. Their responses were often verbose and contained additional or unnecessary details that diluted accuracy. Future work could address this limitation by testing different parameterizations or exploring various prompt engineering strategies.

Specifically, GPT-3.5 and GPT-4o showed the highest performance in *answer relevance*, reflecting their ability to retrieve and synthesize appropriate answers based on the query. In contrast, Claude exhibited superior *faithfulness*, suggesting greater alignment with the clinical guidelines and fewer hallucinations or inaccuracies. These findings underscore the importance of selecting models that balance computational efficiency with clinical reliability in retrieval-augmented generation-based applications for obstetric care.

The Mistral model, for its part, proved to be the most consistent in terms of *faithfulness* across both judges, but performed poorly in *answer relevance*. This result suggests that while its answers were faithful to the context, they were not always directly relevant to the question, likely due to retriever limitations. In contrast, GPT-3.5 and GPT-4o achieved better results in *answer relevance*, possibly by compensating for gaps in retrieved content with their pretrained general knowledge. This behavior, however, should be further evaluated to ensure strict adherence to clinical guidelines.

The retriever performance was a key limitation, as evidenced by the *context recall* and *context precision* metrics. *Context recall* remained at 0.5 or lower for most models, except Claude 3.5 (0.63) and GPT-4o (0.55), when judged by GPT-4o. This indicates that relevant documents or fragments were not consistently retrieved, which limits the quality and completeness of the answers. Similarly, *context precision* was below 0.39 across all models, indicating that the retriever often returned irrelevant or only partially useful content. This lack of precision reduces the likelihood of generating accurate answers, since the retrieved documents may not contain the necessary details. These results highlight the need for future research to focus on improving retrievers, particularly through enhanced embedding models and more robust similarity assessment methods.

Despite the valuable insights of this study, several limitations should be considered. First, the evaluation was based on a relatively small set of clinical questions (n = 10). Consequently, the findings should be interpreted with caution, as they may not fully capture model performance across a broader range of clinical scenarios. Nevertheless, this limited set of questions was appropriate for a pilot study and provided preliminary evidence on the capacity of evaluation models to generate maternal healthcare responses aligned with clinical guidelines.

Another limitation is that only one medical expert was involved in defining the ‘true’ responses from the guideline, which prevented measurement of inter-rater reliability. To reduce potential bias, the task design aimed to minimize subjectivity by directly grounding the answers in the clinical guidelines. In addition, this study assessed model performance in conjunction with the retrieval component, without independent evaluation of retrieval quality. Furthermore, models were tested without systematically varying their configurations, which may have influenced the results. Future research, therefore, should incorporate a larger set of questions, multiple evaluators, independent retrieval assessment, and diverse model configurations to improve the generalizability of the results.

Although artificial intelligence represents a promising tool in clinical practice, including obstetrics, it should not be completely trusted without a critical analysis of its limitations. Its role is to assist, not replace, clinical expertise, since artificial intelligence models remain prone to errors, especially when dealing with complex or nuanced cases, and thus require further validation. The use of large language models in retrieval-augmented generation applications for extracting information from clinical guidelines offers substantial value in terms of efficiency and decision-making. However, several clinical risks and ethical challenges must be addressed. Although such models can provide evidence-based recommendations, final decisions must remain with specialists, as large language models lack the ability to fully interpret patient-specific clinical contexts. The risk of misleading or inaccurate responses persists, as models may generate information that, while grounded in guidelines, may not be directly applicable to individual cases.

From an ethical standpoint, the use of these models also raises concerns regarding patient data privacy, particularly when information is processed in uncontrolled environments. Therefore, it is essential to ensure that retrieval-augmented generation systems function strictly as support tools rather than substitutes for human judgment, with robust security protocols in place to protect sensitive information, while consistently respecting both the autonomy of healthcare professionals and patient confidentiality. Furthermore, although artificial intelligence can rapidly process large datasets and generate useful insights, it cannot replicate the contextual understanding and ethical judgment of a trained clinician. Consequently, this study underscores the need to integrate artificial intelligence outputs with human expertise.

In conclusion, this pilot study assessed the ability of various language models to generate responses based on a Spanish-language clinical guideline in gynecology and obstetrics. The findings suggest that large-scale models outperform mid-scale ones in this task, exhibiting encouraging levels of response relevance. In the best-performing model, the correct explanation was retrieved and included in the answer, as confirmed by expert evaluation. However, expanding the set of questions is required to enhance the statistical robustness of the findings. Moreover, the results highlight the importance of further improving and assessing the retrieval component to ensure stricter adherence to clinical guidelines.
